# Complications of Fixed Full-Arch Implant-Supported Metal-Ceramic Prostheses

**DOI:** 10.3390/ijerph17124250

**Published:** 2020-06-14

**Authors:** Ignacio Gonzalez-Gonzalez, Hector deLlanos-Lanchares, Aritza Brizuela-Velasco, Jose-Antonio Alvarez-Riesgo, Santiago Llorente-Pendas, Mariano Herrero-Climent, Angel Alvarez-Arenal

**Affiliations:** 1 Department of Prosthodontics and Occlusion, School of Dentistry, University of Oviedo, C/. Catedratico Serrano s/n., 33006 Oviedo, Spain; ignaciog.gonzalez@gmail.com (I.G.-G.); ildarionhector@hotmail.com (H.d.-L.); joseanari@gmail.com (J.-A.A.-R.); arenal@uniovi.es (A.A.-A.); 2 Private Practice, 33004 Oviedo, Spain; llorentesantiago@gmail.com; 3 Private Practice, 4150-518 Porto, Portugal; dr.herrero@herrerocliment.com

**Keywords:** fixed prostheses, full-arch prostheses, metal-ceramic prostheses, dental implant, mechanical complications, biological complications

## Abstract

We aimed to assess the biological and mechanical-technical complications and survival rate of implants of full-arch metal-ceramic prostheses, during five years of follow-up. 558 implants (of three different brands) retaining 80 full-arch metal-ceramic prostheses were placed in 65 patients, all of whom were examined annually for biological and mechanical-technical complications during the five years of follow-up. Descriptive statistics and univariate logistic regression were calculated. The cumulative survival rate of the implants was 99.8%, and 98.8% prosthesis-based. Mucositis was the most frequent of the biological complications and peri-implantitis was recorded as 13.8% at restoration-level, 16.9% at patient level and 2.0% at implant level. An implant length greater than 10 mm was shown to be a protective factor against biological complications. The mechanical-technical complications were associated with implant diameter, abutment/implant connection and retention system. Loss of screw access filling was the most frequent prosthetic complication, followed by the fracture of the porcelain. Full-arch metal-ceramic prostheses show a high prevalence of implant and prosthesis survival, with few biological and mechanical-technical complications.

## 1. Introduction

It is well known that the loss of all the teeth of one or both dental arches causes important anatomical, functional, aesthetic and psychological changes, which significantly reduce the quality of life and comfort of patients. Until not many years ago, treatment with a removable complete denture based on principles of physical-chemical and biomechanical retention was the only solution. However, the benefits obtained were not sufficient to guarantee the satisfaction and comfort of the patients, especially those whose alveolar ridge is of low height and poor retentive capacity. The predictability, good prognosis and high survival rates of dental implants seem to be the ideal solution to ensure the stability/retention of the complete prosthesis and the satisfaction of patients in cases of total edentulism. Both overdentures and fixed full-arch metal-porcelain or metal-acrylic resin (hybrid) prostheses, with or without cantilever, are possible solutions. Restorations of this kind have shown high levels of satisfaction and comfort, quality of life and functionality in clinical studies compared to a conventional complete prosthesis [1,2,3].

Although overdentures are the most economical solution of all possible types of implant-supported prostheses to restore all the teeth of an arch, the best functional, aesthetic and comfort/ satisfaction results are achieved with fixed full-arch prostheses, whether they be of metal-porcelain or metal-resin (hybrid prosthesis) [3,4,5,6].

The full-arch screw-retained hybrid prosthesis was conceived and designed to avoid compromises of bone availability or of important anatomical structures (maxillary sinus, inferior alveolar nerve). Since its inception, numerous clinical and laboratory studies have been conducted to evaluate the influence of various factors on peri-implant bone loss, implant survival rate and mechanical-technical complications of restorations, with good results having been obtained regardless of whether the hybrid prosthesis (metal-acrylic resin) was retained by 4, 6 or 8 implants. In general, implant survival rates have been reported, independently of the arch, from 92% to 95.1% at ten years [7,8,9,10]; and up to 100% at five to seven years [10,11,12]. The cumulative survival rate of the prosthesis is similar to that of the implants [10,11,12,13]. In contrast, mechanical-technical complications are frequent, with high percentages that increase with years of use [13,14,15,16]. In addition, these full-arch screw-retained metal-acrylic/resin prostheses have other limitations such as periodic removals for cleaning, loss of initial aesthetics due to wear, and progressive deterioration of the material of the teeth and the prosthetic base, among others, limitations that can be avoided with other types of restoration such as porcelain free metal or metal-ceramic restorations.

Currently, advances in surgical techniques, techniques and materials of guided bone regeneration, predictability of osseointegration and clinical evidence of good results with inclined, narrow or short implants, have made it possible, in great measure, to overcome the anatomic limitations and problems of bone availability in both arches [17,18,19]. In accordance with the foregoing, it is possible, in most clinical situations, to design a fixed full-arch prosthesis in metal-porcelain retained by six or more implants. These restorations, although poorly documented for an edentulous jaw, provide greater functionality and aesthetics, which are very often demanded by patients. However, very few clinical studies have been carried out to evaluate the implant survival rate and mechanical-technical and biological complications of restorations of this kind compared to the large number of studies on resin veneered prostheses. Thus, for fixed full-arch metal-porcelain prostheses, implant survival rates of between 95.0% and 98.7% [6,20,21,22], have been reported for one arch or the other and for different numbers of years of loading. As in full-arch screw-retained metal-acrylic/resin prostheses, mechanical-technical complications of full-arch metal-porcelain restorations are very frequent in a range of 33.3 to 36% at ten years [6,20], although after three years of loading, 88.9% of complication-free restorations have been reported [21] Regarding biological complications, a recent study that combined full-arch metal-ceramic and metal-resin prostheses reported 73.3% of prostheses and 90% of implants as being periimplantitis-free [22].

In relation to the aforementioned, a lack of information in the dental literature regarding the clinical results of implant-supported full-arch metal-porcelain prostheses is verified. This information is necessary for dentists to be able to design restorations of this type with greater predictability. Therefore, clinical studies with larger sample sizes, for one arch and the other, are needed (existing ones do not exceed 55 cases and are usually focused on the maxilla, and in some studies the arch is not specified) [20]; studies carried out in the conditions of a normal clinical practice are also required, without inclined implants or special implant designs [21] and without the drawbacks of immediate loading [6]. Also necessary are studies focused on full-arch metal-porcelain restorations not combined with other types of restorations in the same study [21,22]. 

The objective of this clinical study during the five-year follow-up period was therefore to assess the cumulative survival rate of implants and prostheses, as well as the biological and mechanical-technical complications of fixed full-arch metal-porcelain prostheses. It was also to point out and compare the influence of other independent variables.

## 2. Materials and Methods 

This study is focused on the evaluation of 80 full-arch implant-supported metal-porcelain prostheses placed in 65 patients during five years of follow-up. All patients were checked every year for at least five years. Prosthetic treatment and follow-up of all patients included in the study was performed in a private urban clinic in the period between January 2002 and June 2017. The first prosthesis was made in 2002 and the last one included in the study in 2012, the follow-up thus ending in 2017.

### 2.1. Selection of Patients and Implants 

The criteria for inclusion of patients in the study were: (1) males and females older than 18 years with one or both arches fully edentulous or with any tooth that had to be extracted, (2) having or with the possibility of obtaining sufficient bone height and width to allow the insertion of implants of at least 8 mm in length and 3.3 mm in diameter, (3) having the economic means to meet the cost of treatment, (4) having the capacity to understand and sign an informed-consent form.

Exclusion criteria were: (1) poor general health due to systemic diseases that may compromise or contraindicate implant surgery, (2) previous treatments or taking medications that can seriously influence implant osseointegration, (3) poor oral hygiene conditions and lack of motivation for dental hygiene. This last condition was verified by the examiner in the remaining teeth of the antagonistic arch after periodontal treatment (prophylaxis and motivation for dental hygiene, tartrectomy and, if advisable, scaling and root planing) that must be done of necessity to patients who are going to carry a full-arch implant-supported prosthesis.

Fixed full-arch metal-ceramic prostheses are complex restorations that usually require major surgery, normally with the need for some bone regeneration technique. The foregoing, in addition to the need to avoid possible surgical complications derived from the patient’s previous health conditions, form the basis of the aforementioned exclusion criteria. Systemic diseases are those that may compromise the implant osseointegration, the surgical technique or the patient’s health during the intervention (history of thrombo-embolism; anticoagulant treatment; taking bisphosphonates; diseases that have an impact on bone quality of the jaws; valvular heart problems; history of radiotherapy in the cranio-cervical region; history of immunosuppressive treatment; coagulation abnormalities due to liver or other disease; psychiatric or psychological diseases that make treatment difficult to understand). However, only patients with a history of taking bisphosphonates or who have had radiotherapy in the cranio-cervico-facial region or immunosuppressive treatment and those with significant cardiovascular problems were excluded. Furthermore, it is generally recognized that the presence of periimplantitis, associated or not with an excessive occlusal load, is the main cause of post-load peri-implant bone loss and implant failure. In an extended and complex treatment, such as a full-arch metal-porcelain prosthesis, periimplantitis should be prevented or its presence minimized. Motivation and control by the professional are necessary since periimplantitis and oral health in general depend largely on the hygienic habits of the patient. Thus, prior to surgery, all patients who accepted the estimate for a planned prosthesis treatment, regardless of the periodontal treatment to be performed, were motivated and instructed in oral hygiene techniques. Those who did not have a good level of hygiene were excluded from the study and were given another form of treatment.

The sample thus selected came from the original patient group. This comprised fully or partially edentulous patients (those with teeth in poor condition that had to be extracted) in any dental arch who went to a dental clinic in the period from 2002 to 2012. From this group the patient study group was selected based on the inclusion/exclusion criteria already referred to above. The remaining fully edentulous patients were treated with other types of prosthetic restorations that were not a full-arch metal-ceramic prosthesis. In the reference period, 239 fully edentulous patients (in one or both arches), or those with remaining teeth that had to be extracted, requested treatment. 11 patients did not attend or rejected the proposed treatment for different reasons. The distribution of the remaining patients (228) according to the type prosthetic treatment was as follows: 65 patients received 80 full-arch metal-porcelain prostheses; 20 patients received 20 hybrid or full-arch metal-resin/acrylic screwed prostheses; 112 patients received 194 overdentures (30 in a single jaw arch and 82 in both dental arches); 31 patients received 59 conventional complete prostheses (five patients in a single arch and the rest in both). In addition, another seven conventional complete prostheses corresponded to seven patients with overdenture in the antagonistic arch. No random selection was made to assign patients to one or other group of prosthetic treatment, since the inclusion/exclusion criteria determined the selection of patients in this study. Therefore, those fully edentulous patients who did not meet these conditions received another type of treatment. Thus the distribution of patients/prostheses/implants included in the study was 65/80/558 from the original group of fully edentulous patients treated in the clinic during 2002–2012. The following 163/271/672 were excluded for not complying with the criteria already presented, or 173/271/672 if patients who did not attend or who declined any prosthetic treatment are included.

A total of 558 implants of different brands and characteristics were used (Straumann SLA Tissue Level; Astra-Dentsply OsseoSpeed TX; and Biomet 3i Osseotite External Connection Implant System). An experienced surgeon with one specific implant surgical protocol inserted all of them. In most edentulous jaws, six to eight implants were inserted (only 10 implants were placed in one upper jaw). The number of implants inserted was based on the predetermined treatment plan, depending on the opposing dentition, bone quality and availability, parafunctional habits, bone regeneration or not, morphological characteristics of the edentulous jaws and other force factors. In most patients’ dental arches, some kind of bone regeneration technique was needed (52, 80.0%). These techniques were, preferably, sinus lift, particulate graft, and on-lay grafts. The implants were loaded after three months of osseointegration in the maxilla and after two months in the mandible or when the Implant Stability Quotient (ISQ) was greater than 60.

### 2.2. Prosthetic Protocol and Follow-Up Examinations

During the osseointegration period, the patients used temporary complete acrylic-resin dentures that were modified every month with a soft denture re-liner (Ufigel SC, Voco GmbH, Cuxhaven, Germany) and occlusal control for a balanced bilateral occlusion. The definitive restorations were provided in accordance with the usual technique and systematics of an implant-supported prosthetic rehabilitation. Thus, after the impression was taken, the casts were obtained in type IV plaster (GC FujiRock EP, GC Europe, Leuven, Belgium). The working casts were then transferred to and assembled in a semi-adjustable articulator (Artex® CR. Amann Girrbach AG, Koblach, Austria), the upper one with facial bow and the lower one with a register of Centric Relation in accordance with the established vertical occlusion dimension and with the established aesthetic and functional registers (diagnostic waxing tests). The accuracy of the transfer of the implant position to the work model was checked by means of a verification jig. Subsequently, tests were carried out on the metallic framework to check radiographically and clinically its passive fit using the Sheffield fit test. All metallic frameworks were made in chrome-cobalt alloy and most were cast, except six that were milled. The color was selected. The occlusion of the porcelain-fused metal was checked in the biscuit phase after seating the prosthesis in the mouth. Once the prostheses were finished, 46 were screwed into place in accordance with the manufacturers’ recommendations (35 of one piece, five of two pieces and six of three pieces) and 34 (12 of one piece, 16 of two piece, six of three pieces) were cemented with a temporary urethane-based resin cement (Stone Implant, Sweden & Martina SpA, Due Carrare, PD, Italy). The cemented system was chosen when there was dis-parallelism between the implants. All screwed prostheses were placed with an intermediate abutment with the exception of three, which were screwed directly to the implant and were made by CAD-CAM. The occlusal scheme of all the prostheses was mutually protected occlusion, achieved by occlusal adjustment in the articulator and in the patients’ mouths. A single prosthodontist, assisted by a dental hygienist, prepared all the prostheses and examined the patients during routine recall visits, emergency, and check-up appointments. All patients were requested to go for six-monthly check-ups in the first year, and thereafter at least once a year. During the annual check-ups, occlusion was recorded using 8 µm articular paper (Arti-Fol. Bausch Articulating Paper Inc., Nashua, NH, USA) and each patient was given a thorough visual examination, periodontal probing and periapical radiographs to detect biological complications of the implants and mechanical-technical complications of the prostheses. All patients received annual supportive periodontal therapy, every six months in the case of patients with mucositis, until they corrected their hygiene habits. In addition, the prostheses were disassembled every two years, except in cases of intense inflammatory signs, for more effective hygiene. All patients in the study group were followed-up each year for five years (some patients have been followed up for more than five years). No patient abandoned the treatment or died during the five years of follow-up, perhaps because the significant economic cost of the treatment was good reason for non-abandoning it.

### 2.3. Assessment Criteria 

Implant survival was defined as the functional use of implants without removal for any reason. These implants satisfy the classic success criteria formulated by van Steenberghe [23]. Conversely, implants that were removed for any reason were assessed as failures. An implant was considered compromised or of doubtful prognosis if it suffered progressive peri-implant bone loss greater than 3 mm but did not have to be removed. A prosthesis was considered a survivor if it was functional even if some mechanical-technical complication required attention. Failure was considered if the prosthesis had to be replaced for any reason. Biological complications included mucositis and periimplantitis. Mucositis was diagnosed by the presence of inflammation or suppuration of soft peri-implant tissue with bleeding on probing, associated with a pocket depth of less than 5 mm and no bone loss around implants. Periimplantitis was recorded when there was bleeding on probing associated with a pocket depth greater than or equal to 5 mm and radiographic peri-implant bone loss that was greater than 2 mm. Although the diagnosis of periimplantitis and the amount of associated bone loss are a matter of controversy, the criteria used are equivalent to those reported in the literature [23,24,25,26].

Mechanical-technical complications included the mechanical ones related to the prefabricated components and caused by mechanical forces (fracture of implants, abutments or screws and loosening/loss of any screw) and the technical ones related to the components manufactured by the laboratory or the clinician (fracture/chipping of the porcelain, fracture of the framework, de-cementation of any retainer and loss of the closing material of the access hole to the prosthetic screw). Any fracture of the porcelain was recorded, regardless of the classification and severity criteria reported by Anusavice’s study [27]. Likewise, a complication was considered to be minor if it required 60 minutes or less of chair time to correct and major if more time was required or if the element had to be sent to the dental technician´s laboratory. In clinical practice, these periods are realistic, also being cited by other authors [28].

### 2.4. Statistical Analysis 

In descriptive statistics, the mean value and the standard deviation were calculated for quantitative variables, and absolute and frequency distribution were calculated for qualitative variables. The comparison between variables was done using the univariate logistic regression analysis. A multivariate analysis was assessed, but a model could not be identified with the study data. The odds ratio (OR) and 95% confidence interval (CI) were calculated. The cumulative survival rates of the implants and of the prostheses were estimated by means of Kaplan-Meier analysis. A data matrix created ad hoc with the Excel 2013 spreadsheet (Microsoft Co., Redmond, WA, USA) was used to collect the data. Statistical analysis was carried out using the software package Stata v.13 (StataCorp LLC, College Station, TX, USA). The level of statistical significance was considered at a p-value of less than 0.05.

## 3. Results

### 3.1. Distribution of Patients, Implants and Prostheses

Sixty-five patients (46 females and 19 males) aged between 39 and 80 years (average age 58.7) were enrolled in this prospective clinical study. 40 patients were younger than 60 and the rest between 61 and 80 years old. The average age of the men was 63.7 years (range 52 to 80 years) and of the women 56.5 years (range 39 to 79 years). The great majority of the patients had a history of periodontal disease (92.3%) which, added to other causes, had resulted in the loss of all the teeth of one or both of the arches. 52.3% of the patients acknowledged grinding or clenching their teeth and only 24.6% of cases were smokers of more than ten cigarettes a day. 80% of patients required some technique of bone regeneration before the implants were inserted.

A total of 558 implants of different brands (410 Straumann SLA Tissue Level; 133 Astra-Dentsply OsseoSpeed TX; and 15 Biomet 3i Osseotite External Connection Implant System) were inserted, 438 in the maxilla and 120 in the jaw. The number of implants and their locations in the edentulous jaws, as well as their geometric characteristics and connection type, are shown in Table 1, Table 2, Table 3, Table 4, Table 5, Table 6, Table 7, Table 8 and Table 9.

In total, 80 fixed full-arch metal-porcelain prostheses were manufactured and installed, 60 in the maxilla and 20 in the mandible. Both arches were rehabilitated in 15 patients. In the opposing dentition, the rest of the patients had: natural teeth, natural teeth in combination with conventional fixed partial metal-porcelain prostheses, natural teeth in combination with fixed partial metal-porcelain implant-supported prostheses, or acrylic-resin dentures. The majority of the full-arch prostheses (30 upper and 17 lower) had the implants splinted in a single section, i.e., they were one-piece prostheses. The remaining 33 fully edentulous arches received a full arch treatment which consisted of 21 prostheses divided into two sections at the midline (19 in the maxilla and two in the mandible) and 12 prostheses (11 in the maxilla and one in the mandible) divided into three sections (one anterior and two posterior). Greater ease of obtaining a passive fit in dis-parallel implants, the unfavorable morphology of the anterior sector of edentulous jaws, minimizing the mandibular deflection phenomenon and the possibility of removing only one section if a complication is suspected, are the arguments for the described division. Other authors also support this division [29,30,31] Regardless of the division into sections, all restorations were designed with 12 teeth per arch, with an average of 4.9 pontics and without cantilevers in the majority (63) or with a mesial or distal cantilever of short extension, similar to the mesial-distal length of one tooth (three in each arch) or two teeth (five in each arch), and only one with a cantilever of more than two teeth (in the mandible). Table 1, Table 2, Table 3, Table 4, Table 5, Table 6, Table 7, Table 8 and Table 9 show other characteristics of the prostheses in relation to their distribution in the edentulous jaws, the number of implants for each prosthesis, the crown/implant ratio, the retention system, and the opposing dentition.

### 3.2. Cumulative Survival Rate of the Implants

**Figure 1:**
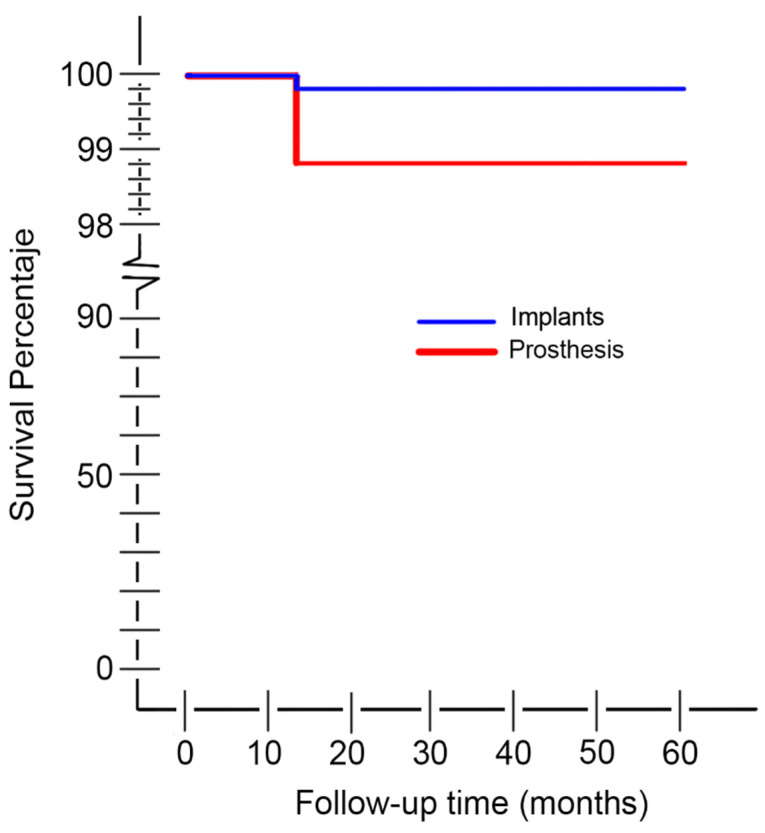
Kaplan-Meier curve for survival estimate of implants and prostheses.

At the end of the analysis period (June 2017), one of 558 implants was removed in one male patient. This was done due to fracture after 13 months of loading. The 3.3 × 12 mm implant (Straumann SLA) was located in the incisal area of the maxilla of a three-section full-arch prosthesis. The patient (56 year old) was a non-smoker with a history of periodontitis and bone regeneration, a bruxist with natural dentition in the antagonist arch; he also had Angle’s class III malocclusion, which was rehabilitated with an edge-to-edge occlusion prosthesis at maximal intercuspal position. The cumulative survival rate was subsequently 99.8% at implant level, 98.5% at patient level and 98.8% at prosthesis level (Table 10, Table 11 and Figure 1).

However, 11 implants with pockets greater than 5 mm and with more than 2–3 mm of peri-implant bone loss were registered in some implants due to periimplantitis. Although they were not needed or were not removed due to the treatment performed, these implants can be considered of doubtful prognosis and with a possible future failure. Thus, considering only this item, the cumulative rates of implants of good prognosis without periimplantitis were 98.0% at implant, 83.5% at patient and 86.2% at prosthesis-level.

### 3.3. Biological Complications

During the five-year follow-up period of the restorations, the percentage of biological complications was 81.3% (65/80 restoration-based). The majority were mucositis (54), whereas periimplantitis only occurred in some implants of 11 prostheses (13.8% restoration-level), with a prevalence of 16.9% at patient level and 2.0% at implant level.

The majority of patients with periimplantitis were women (nine) of 56.6 years of average age. Nine patients were non-smokers, six were bruxists, seven had needed bone regeneration and all had been diagnosed with previous periodontitis. Regarding the implants, all were of internal connection and predominantly greater than 10 mm in length (seven). In addition, periimplantitis occurred more frequently when the crown/implant ratio was equal to one (seven) and in standard diameter implants (six). The largest number of these implants were in the maxillary arch (10), all of them in the incisal area. At the level of the prostheses, periimplantitis was mainly located in the maxillary arch (10) and with natural teeth in the opposing dentition (nine). In addition, none of the prostheses had cantilevers; those divided into one section (six cases) predominated over those divided into two sections or three sections (four and one cases, respectively) and those retained by eight implants (eight cases). Likewise, periimplantitis was somewhat more frequent in cemented prostheses (seven) compared to screwed ones (4), while mucositis was more frequent in screw-retained prostheses (31). Chronologically, all periimplantitis was recorded after the fourth year.

The univariate logistic regression analysis shows that of all the independent variables in this study (patient, prosthesis and implant characteristics), only the location, the length and the implant brand significantly influenced the appearance of mucositis either as a risk factor (implant brand, OR = 1.76) or as protection factors (location and length) with OR less than 1 (Table 1, Table 2 and Table 3). Likewise, of all the variables, only the implant length greater than 10 mm was shown to be a significant protective factor for periimplantitis, with an OR of 0.13. For the other variables, no statistically significant relationship was found (Table 4, Table 5 and Table 6).

### 3.4. Mechanical-Technical or Prosthetic Complications

After five years of follow-up, 38.8% (31/80 restoration-based) of the restorations and 47.7% (31/65 patient based) of patients presented some mechanical-technical complication or other. Table 7, Table 8 and Table 9 show that the greatest risk of the presence of these complications is significantly related to the retention system (screwed), the implant/abutment connection type (external) and the implant brand. However, an implant diameter greater than 4.1 mm and smoking more than ten cigarettes/day would be significant protective factors.

Table 12 shows the type and distribution of mechanical-technical complications and their relationship with the prosthetic retention system. Most of these complications were the fall of closure material from the hole giving access to the prosthetic screw (13), fracturing of the porcelain (10) and the loosening of a trans-occlusal or prosthetic screw (four) and, to a lesser extent, the de-cementation of the restoration and the fracturing of a screw, implant or metal framework (one of each). Each type of mechanical-technical complication was more frequent in screwed prostheses compared to cemented ones, with statistically significant differences when compared globally.

### 3.5. Survival of Restorations and Repair Time

During the follow-up time, only the anterior segment of a single maxillary prosthesis divided into three sections had to be replaced due to the fracture of an implant (Table 11). On the other hand, most complications, both biological and mechanical (53/80 prosthesis level), required less than 60 minutes of chair time for repair, 13 required prolonged periodontal treatment while eight elements were sent to the prosthetic laboratory.

## 4. Discussion

This study shows, at five years follow-up, the results obtained with of 80 implant-supported fixed full-arch metal-porcelain prostheses fitted in 65 patients. The analysis of restorations of this kind has rarely been reported in the literature; only two recent studies report the results of 55 similar prostheses at five years [22] and 36 in the short-term, at two-year follow-up [21]. In addition, the variability in the years of follow-up, number and characteristics of the implants, design of the restoration and combination in the same study of prostheses of different materials, make it difficult to compare the data obtained with those of other studies [6,12,20,21,22,32,33].

### 4.1. Implant and Restoration Survival Rates

Compared to previous studies of full-arch metal-porcelain prostheses, the implant survival of this study (99.8%) is similar to the 99.1% found at two years of average functioning [21] and a little higher than the survival rate from other studies at five-year [22] and ten-year follow-up [6,20]. At the same time, it is not very different from the survival rate in full-arch metal-resin prostheses supported by five-six or four implants of the "all on four" protocol. In these, implant survival percentages are reported over a range of 98.7% to 100%, depending on the prosthetic design and on whether the loading period is five years or more [7,10,11,34]. For longer follow-up periods (36 years) and with different prosthesis designs, Chrcanovic et al [35] have reported implant survival rates of 87.8%. On the other hand, Jemt [36], with different designs of metal-resin prostheses, reports the long-term influence of the implant surface and location in the edentulous jaws on the implant survival rate. In the 1986–2002 period, the survival rate was 75.7% and 96.4% for maxilla and mandible respectively. However, with the use of rough surface implants in the period 2002–2015, this difference was reduced, with survival rates of 91.9% in the maxilla and 96.2 in the mandible.

The replacement in this study of only the anterior section of one restoration shows the favorable result for full-arch metal-ceramic prostheses (98.8% survival), similar to that reported in the literature. The study of Papaspyridakos [22] (55 metal-ceramic prostheses) indicated that on average 98.1% survived after five years of functioning. In other studies with fewer restorations (between 20 and 36), 100% survival rates are mentioned for short-term and long-term loading periods [6,12,20,21,32]. In this respect, the survival data of full-arch metal acrylic-resin prostheses provided by the literature show a variable survival rate that depends on the observation time, with prevalences of 100% at 5 years in some studies [7,10,14,33] decreasing to 93.3% [11] and to even lower values after ten years of follow-up [3,9]. Summing up, both the full-arch metal-ceramic prostheses of this study and the metal-resin ones, based on the literature data, do not seem to have an important influence on the survival of the implants and restorations.

These high survival rates endorse the importance of an effective periodontal care support, together with achieving a prosthetic design that allows a greater ease of hygiene, an adequate occlusal scheme and control of parafunctions.

### 4.2. Biological Complications

However, the present study is not free from complications. During the observation time, the restoration-related prevalence of biological complications (mucositis and periimplantitis) was 65/80 restoration-based, with five times more cases of mucositis (54) compared to periimplantitis (11). These findings are in line with those reported in previous systematic reviews and the general consensus [37,38]. The exclusion of patients with systemic diseases or with low motivation and poor oral hygiene in the antagonistic teeth could have influenced the results obtained. Fixed full arch metal-porcelain prostheses are difficult and complex to maintain, with worse prognosis of biological complications in these patients. However, the estimation of the prevalence of periimplantitis should be taken with caution due to the lack of uniformity in the definition, the variability in the design of the restorations, in the number and characteristics of the implants, in the evaluation time, in the design, selection and sample size of the clinical studies. The prevalence of periimplantitis in this study is somewhat higher than the results provided by other studies of full arch and protocol "all on four" of metal porcelain prostheses [6,12]. By contrast, it is much lower than implant-based and patient-based periimplantitis data cited in similar metal-acrylic resin prostheses in some studies [7,39,40] or in combination with metal-ceramic prostheses from the Papaspyridakos study [22]. The supportive periodontal therapy every year in addition to the systematic removal of prostheses every two years for better cleaning and periodontal therapy may explain the low percentage of periimplantitis findings. By contrast, the periimplantitis data most often cited in the literature in fully edentulous patients treated with a metal-acrylic resin prosthesis are related to greater difficulty for the patients in maintaining oral hygiene. This is due to a greater contact area of the acrylic-resin with the mucous membrane of the alveolar ridge (even if there is a slight separation rather than contact) and also to alterations arising from the deterioration of the acrylic-resin (staining, cracks, breaks, etc.). All of this may lead to a greater increase in bacterial plaque and the presence of periimplantitis.

The increased risk of mucositis is only significantly related to one of the variables in this study, namely, the brand of implants used (and the associated surface treatment they have). Those implants that had no sand-blasted, large grit, acid-etched (SLA) surface show a 1.76 times greater risk. Factors related to biofilm onset [41], differences in the transmucosal portion (tissue level versus bone level) [42,43] and differences in surface roughness [44,45] could explain the result. Likewise, the logistic regression analysis did not show any risk factors related to patients, prostheses, or implants for periimplantitis. On the other hand, it showed a significant protective effect of long implants (greater than or equal to 10 mm) for the presence of both mucositis and periimplantitis. This data supports the clinical choice of long implants to reduce the risk of periimplantitis and prevent implant failure after loading. Although this “per se” protective effect can be controversial, since implants with a length equal to or greater than 10 mm are placed where there is more bone available and possibly less bone resorption before surgery, this bone favorability may result in less failure. However, scientific evidence based on systematic reviews of randomized controlled clinical trials, with and without meta-analysis, shows mismatched results, with no difference in rates of success or failure between short (less than 10 mm) and long (equal to or greater than 10 mm) implants [46,47,48]. Likewise, another review [49] concludes that most studies show significantly reduced survival with implants less than 10 mm in length compared to longer ones (10 mm or more). Similarly, a lower chance of survival and higher failure rate of short implants [50,51]. are reported and a recent consensus revealed a lower survival rate of short implants (6 mm or less) compared to standard length ones [19]. However, the aforementioned data are not exactly comparable to those in the present study.

On the other hand, although the data of this study indicate the non-relationship of the risk of periimplantitis to the location of the implants in the edentulous jaws, when only the 11 cases of peri-implantitis at restoration-level are taken into account, a higher frequency is recorded in the maxilla and in its incisal area. This can be explained by the greater total number of implants compared to those in the mandible, and also by the predominance of splinted prostheses in a single section that could have made the removal of bacterial plaque difficult. Another possible explanation is the anatomical characteristics of vascularization, bone quality and direction of the occlusal load in the maxilla and its incisal area. Clinical studies [52], previous systematic reviews and meta-analysis [47,50] report a greater risk to the implants placed in the maxilla than those placed in the mandible. Likewise, the study of Francetti et al. [39] of full-arch rehabilitations, shows a higher rate of implants without periimplantitis in the mandible compared to the maxilla at ten years follow-up.

The highest frequency of periimplantitis both in patients with previous periodontitis and with implant-supported cemented prostheses is sufficiently documented [40,53,54]. However, the results of this study, which show that all cases of periimplantitis occurred in patients with previous periodontitis and also that more periimplantitis was found in cemented prostheses than in screwed ones, should be taken with caution since the logistic regression analysis does not support this association

### 4.3. Mechanical-Technical Complications 

This study revealed that the greatest risk of mechanical-technical complications is significantly associated with the following variables: the restoration retention system (screwed), the implant connection kind (external connection) and the brand of the implants (non-SLA). A diameter larger than 4.1 mm would be a significant protection factor favoring a lower rate of prosthetic complications. The greatest risk of external connection is consistent with that reported in clinical studies and previous systematic reviews [55,56]. Perhaps this can be explained by the worse adjustment of the abutment to the external connection compared to the internal one [57] and by a greater magnitude of micro-movement in external connection under load, which influences the preloading of the screws [56].

The increase in the functional surface area with wide diameter implants can influence the moment of inertia to resist bending, transmitting less stress to the prosthesis/implant/bone complex. These facts may explain the protective effect of these implants. In agreement with this effect, Pieri [58] in a five-year follow-up reported a significantly higher risk of prosthetic complications with narrow diameter implants (less than 3.3 mm) compared to standard ones (4–4.5 mm) in fixed partial dentures of maxilla and jaw posterior sectors. In addition, a consensus of 2018 states in its recommendations that reducing the implant diameter increases the risk of implant or component fracture [19]. The smaller diameter of the screws, with lower preload values and/or lower shoulder margin of the intermediate abutments and also with smaller support surface for the veneering material of the narrow implants, can explain their unsuitability. However, very little attention is devoted to the favorability of wide implants in mechanical-technical complications in the literature.

The highest risk of mechanical-technical complications in screw-retained prostheses (OR = 5.0) is in agreement with previous long-term studies in implant-supported restorations [59,60] and in disagreement with others that have reported a higher non-significant percentage of complications in cemented than in screwed prostheses [61,62].

The evaluation of the distribution of mechanical-technical complications revealed that the loss of the material that closes the access hole to the trans-occlusal screw was the most frequent. Although this complication is reported very little in the literature, in a systematic review, a five-year cumulative rate of 5.4% is indicated in implant-supported fixed dental prostheses [63] yet it is empirically often cited by clinicians. A better sealing of the material and the absence of occlusal contacts in the area should be considered in order to minimize this complication. The fracturing or chipping of the porcelain was the second most frequent complication. Half of chipping/porcelain fracture cases occurred in patients with both dental arches rehabilitated. This data, also recorded in a previous study [12], could be considered a risk factor. The influence of different factors on the fracturing /chipping of the porcelain, the variability in the design and material of the restorations and also the loading time of the different studies found in the literature, make comparison with the data of this study difficult. Nevertheless, the prevalence found is similar to that reported in studies of full-arch metal-ceramic prostheses with a variable number of implants [20,22] but different (higher or lower) in relation to the years of follow-up of other studies [6,21,33]. In contrast, technical complications related to the aesthetic material of full-arch metal-resin prostheses reported in multiple studies [8,11,13,14,15,16] are more prevalent compared to those that occur with the porcelain prostheses in the present study. This is explained by the greater porosity, lower mechanical resistance and the greater influence of time in the deterioration of the aesthetic prosthetic tooth material. The loosening of the abutment or prosthetic screws is a frequent technical complication that in some systematic reviews is cited as the most common technical complication in single crowns and fixed implant reconstructions [64,65,66]. However, in the present study, the loosening of the prosthetic screw was the third most common complication, affecting only 5% of the prostheses (4/80 prosthesis-based). This finding is in agreement with several other studies of implant-supported unitary, partial and fixed full-arch prostheses [12,15,63,64,66] and in disagreement with other studies at ten years of follow-up that report a lower percentage [67] and even with a study that reported no loosening of abutment screws in 19 full-arch metal-porcelain prostheses with a ten-year load [20]. The low rate of screw loosening might partly be explained by better screw quality, by the use of suitable torque/preload and by the tendency toward less screw loosening at internal implant-abutment connections [55,64]. The most recent improvements in the mechanical properties of the screws, based on the design and quality of alloys, may also explain the low percentage of screw fractures (1.25% prosthesis based) compared to previous studies [9].

On the other hand, the present study reveals that most of the complications that occurred in full-arch metal-porcelain prostheses were easily and quickly resolved, the professional requiring less than one hour of chair time to carry out repair work. A similar result is reported in other studies [20].

However, this study is not free from limitations. The most important is the uniformity of the sample provenance. All the patients came from a single dental-surgery and were treated by one and the same professional. Although this may be an advantage since there is no intra-observer variability, it is a limitation as the results cannot have a general projection. The use of three implant brands with different characteristics, and the larger sample employed of one of them, may be another limitation. Non-consideration of the statistical power of this study (1-β), due to the fact that the strategy employed was not intended to make comparisons between groups of patients classified in relation to variables of interest, can be a limitation and could have influenced the results.

## 5. Conclusions

In accordance with the univariate logistic regression analysis carried out, the results obtained and the limitations of studies of this nature, the following conclusions can be drawn:

1. Full-arch metal-porcelain prostheses have a high cumulative survival rate of implants (99.8%) and restorations (98.8%) after a five-year follow-up period.

2. A low prevalence of periimplantitis (16.9% at patient level and 2.0% implant based) was found for this prosthetic design at five-years follow-up. None of the variables related to patients, prostheses or implants were risk factors. Only an implant length greater than 10 mm is shown to be a protective factor.

3. After five-years of follow-up, 38.8% of the restorations and 47.7% of patients presented some kind of mechanical-technical complication. The screwed retention of the prostheses, the external connection of the implants and the non-SLA implants significantly increase the risk of some mechanical-technical complication. In contrast, an implant diameter greater than 4.1 mm is a protection factor.

4. The most frequent complication was the loss of the closure material of the access hole to the prosthetic screw, followed by the fracturing of the porcelain. Most of the complications were minor, requiring less than 60 minutes of chair time for repair.

5. The design of full-arch metal-ceramic prostheses seem to be successful for the rehabilitation of totally edentulous patients. The data from this study can help practitioners to choose implant-supported full-arch restorations made in metal-ceramic, with the security of great aesthetics and good predictability of complications and implant failure, which favors patient acceptance despite their higher economic cost.

## Figures and Tables

**Table 1:** Distribution of mucositis complications in the sample in relation to patient-based variables.

Patients (n = 65) (42/65)			
Variable	OR	(ORCI)	*p*
Sex	0.91	(0.30–2.78)	0.875
Female (30/46) *			
Male (12/19)			
Age Year Group			
<51 (8/12) *			
51–60 (17/28))	0.77	(0.19–2.19)	0.727
61–70 (10/15)	1.00	(0.20–5.00)	1.00
71–80 (7/10)	1.16	(0.19–7.11)	0.367
Previous Periodontitis	1.75	(0.36–10.55)	0.823
No (3/5) *			
Yes (39/60)			
Bruxism	1.32	(0.47–3.65)	0.593
No (19/31) *			
Yes (23/34)			
Smoker > 10 Cigarettes/Day	3.07	(0.80–11.70)	0.100
No (34/49) *			
Yes (8/16)			
Bone Regeneration	2.62	(0.76–0.85)	0.127
No (6/13) *			
Yes (36/52)			

Total of mucositis complications = 54. Univariate logistic regression analysis. Frequency in brackets (* = Reference category; OR = Odds ratio; ORCI= 95% Odds ratio Confidence Interval).

**Table 2:** Distribution of mucositis complications in the sample in relation to prosthesis based variables.

Prosthesis (n = 80) (54/80)			
Variable	OR	(ORCI)	*p*
Site	0.62	(0.19–1.94)	0.411
Mandible (15/20) *			
5 implants (7)			
6 implants (9)			
7 implants (1)			
8 implants (3)			
Maxilla (39/60)			
6 implants (21)			
7 implants (2)			
8 implants (36)			
10 implants (1)			
Sections			
One (30/47) *			
Two (16/21)	1.81	(0.56–5.82)	0.32
Three (8/12)	1.13	(0.29–4.38)	0.85
Without Cantilevers (40/63)	1.92	(0.56–6.56)	0.275
Maxilla (13/52) *			
Mandible (2/11)			
Retention	0.98	(0.38–2.54)	0.981
Cemented (23/34) *			
Maxilla (29)			
Mandible (5)			
Screwed (31/46)			
Maxilla (31)			
Mandible (15)			
Crown/Implant Ratio			
< 1 (10/18) *			
Maxilla (17)			
Mandible (1)			
1 (34/49)	1.81	(0.59–5.50)	0.29
Maxilla (35)			
Mandible (14)			
> 1 (10/13)	2.66	(0.54–13.98)	0.22
Maxilla (8)			
Mandible (5)			
Opposing Dentition	1.11	(0.74–1.66)	0.626
Natural Teeth (27/30) *			
Acrylic/Resin (1/2)			
Metal-Porcelain and Teeth (9/18)			
Metal-Porcelain (17/30)			

Total of mucositis complications = 54. Univariate logistic regression analysis. Frequency in brackets (* = Reference category; OR = Odds ratio; ORCI= 95% Odds ratio Confidence Interval).

**Table 3:** Distribution of mucositis complications in the sample in relation to implant based variables.

Implants (n = 558) (54/558)			
Variable	OR	(ORCI)	*p*
Site	0.57	(0.36–0.91)	0.019
Mandible (15/120) *			
Maxilla (39/438)			
Implant area			
Incisal (32/150)			
Maxilla (119)			
Mandible (31)			
Canine (5/101)	0.98	(0.58–1.68)	0.957
Maxilla (77)			
Mandible (24)			
Premolar (8/167)	1.07	(0.64–1.62)	0.94
Maxilla (126)			
Mandible (41)			
Molar (9/140)	1.02	(0.63–1.66)	0.93
Maxilla (116)			
Mandible (24)			
Implant Diameter			
Narrow (< 4 mm) (19/82) *			
Standard (4–4.1 mm) (25/422)	0.84	(0.50–1.41)	0.516
Width (> 4.1 mm) (10/54)	0.60	(0.29–1.23)	0.168
Length	0.45	(0.20–0.99)	0.048
< 10 mm (20/42) *			
> 10 mm (34/516)			
Connection	0.92	(0.32–2.22)	0.86
Internal (49/543) *			
External (5/15)			
Implant Brand	1.76	(1.14–2.72)	0.011
Straumann SLA Tissue Level (47/410) *			
Others (7/148)			
Astra Osseospeed (133)			
Biomet 3i Osseotite (15)			

Total of mucositis complications = 54. Univariate logistic regression analysis. Frequency in brackets (* = Reference category; OR = Odds ratio; ORCI= 95% Odds ratio Confidence Interval).

**Table 4:** Distribution of periimplantitis complications in the sample in relation to patient-based variables.

Patients (n = 65) (10/65)			
Variable	OR	(ORCI)	*p*
Sex	0.22	(0.026–1.94)	0.177
Female (9/46) *			
Male (1/19)			
Age Year Group			
< 51 (2/12) *			
51–60 (5/28)	1.08	(0.18–6.58)	0.93
61–70 (2/15)	0.77	(0.092–6.44)	0.809
71–80 (1/10)	0.55	(0.042–7.21)	0.653
Previous Periodontitis	0.9	(0.09–8.63)	0.93
No (0/5) *			
Yes (10/60)			
Bruxism	1.44	(0.36–5.69)	0.568
No (4/31) *			
Yes (6/34)			
Smoker > 10 Cigarettes/Day	0.70	(0.134–5.451)	0.674
No (9/49) *			
Yes (1/16)			
Bone Regeneration	0.51	(0.11–2.36)	0.396
No (3/13) *			
Yes (7/52)			

Total of periimplantitis complications = 11. Univariate logistic regression analysis. Frequency in brackets (* = Reference category; OR = Odds ratio; ORCI= 95% Odds ratio Confidence Interval).

**Table 5:** Distribution of periimplantitis complications in the sample in relation to prosthesis based variables.

Prosthesis (n = 80) (11/80)			
Variable	OR	(ORCI)	*p*
Site	3.80	(0.45–31.73)	0.218
Mandible (1/20) *			
5 implants (7)			
6 implants (9)			
7 implants (1)			
8 implants (3)			
Maxilla (10/60)			
6 implants (21)			
7 implants (2)			
8 implants (36)			
10 implants (1)			
Sections			
One (6/47) *			
Two (4/21)	1.60	(0.40–6.42)	0.50
Three (1/12)	0.44	(0.10–1.79)	0.254
Without cantilevers	0.30	(0.03–2.56)	0.275
Maxilla (0/52) *			
Mandible (0/11)			
Retention	0.36	(0.09–1.37)	0.137
Cemented (7/34) *			
Maxilla (29)			
Mandible (5)			
Screwed (4/46)			
Maxilla (31)			
Mandible (15)			
Crown/Implant Ratio			
< 1 (2/18) *			
Maxilla (17)			
Mandible (1)			
1 (7/49)	1.33	(0.25–7.10)	0.73
Maxilla (35)			
Mandible (14)			
> 1 (2/13)	1.45	(0.17–11.93)	0.72
Maxilla (8)			
Mandible (5)			
Opposing Dentition	0.94	(0.551–1.617)	0.834
Natural Teeth (9/30) *			
Acrylic/Resin (1/2)			
Metal-Porcelain and Teeth (0/18)			
Metal-Porcelain (1/30)			

Total of periimplantitis complications = 11. Univariate logistic regression analysis. Frequency in brackets (* = Reference category; OR = Odds ratio; ORCI= 95% Odds ratio Confidence Interval).

**Table 6:** Distribution of periimplantitis complications in the sample in relation to p implant-based variables.

Implants (n = 558) (11/558)			
Variable	OR	(ORCI)	p
Site	2.78	(0.35–21.93)	0.332
Mandible (1/120) *			
Maxilla (10/438)			
Implant area			
Incisal (11/150)			
Maxilla (10/119)			
Mandible (1/31)			
Canine (0/101)	0.24	(0.28–2.03)	0.191
Maxilla (77)			
Mandible (24)			
Premolar (0/167)	0.44	(0.10–1.79)	0.254
Maxilla (126)			
Mandible (41)			
Molar (0/140)	0.16	(0.01–1.40)	0.099
Maxilla (116)			
Mandible (24)			
Implant Diameter			
Narrow (< 4 mm) (5/82) *			
Standard (4–4.1 mm) (6/422)	0.77	(0.16–3.70)	0.747
Width (> 4.1 mm) (0/54)	0.75	(0.60–8.53)	0.820
Length	0.13	(0.36–0.46)	0.002
< 10 mm (4/42) *			
> 10 mm (7/516)			
Connection	2.38	(0.29–19.47)	0.417
Internal (11/543) *			
External (0/15)			
Implant Brand	0.65	(0.14–3.08)	0.59
Straumann SLA Tissue Level (8/410) *			
Others (3/148)			
Astra Osseospeed (133)			
Biomet 3i Osseotite (15)			

Total of periimplantitis complications = 11. Univariate logistic regression analysis. Frequency in brackets (* = Reference category; OR = Odds ratio; ORCI= 95% Odds ratio Confidence Interval).

**Table 7:** Distribution of mechanical technical complications in the sample in relation to patient-based variables.

Patients (n = 65) (29/65)			
Variable	OR	(ORCI)	*p*
Sex	0.86	(0.29–2.54)	0.794
Female (21/46) *			
Male (8/19)			
Age Year Group			
<51 (5/12) *			
51–60 (12/28)	1.05	(0.26–4.13)	0.944
61–70 (8/15)	1.6	(0.34–7.40)	0.548
71–80 (4/10)	0.93	(0.17–5.15)	0.937
Previous Periodontitis	1.13	(0.01–1.24)	0.078
No (5/5) *			
Yes (24/60)			
Bruxism	0.95	(0.36–2.55)	0.933
No (14/31) *			
Yes (15/34)			
Smoker > 10 Cigarettes/Day	0.196	(0.049–0.78)	0.021
No (26/49) *			
Yes (3/16)			
Bone Regeneration	0.628	(0.18–2.13)	0.456
No (7/13) *			
Yes (22/52)			

Total of mechanical-technical complications = 31. Univariate logistic regression analysis. Frequency in brackets (* = Reference category; OR = Odds ratio; ORCI= 95% Odds ratio Confidence Interval).

**Table 8:** Distribution of mechanical technical complications in the sample in relation to prosthesis based variables.

Prosthesis (n = 80) (31/80)			
Variable	OR	(ORCI)	*p*
Site	1.00	(0.36–2.76)	1.00
Mandible (7/20) *			
5 implants (7)			
6 implants (9)			
7 implants (1)			
8 implants (3)			
Maxilla (24/60)			
6 implants (21)			
7 implants (2)			
8 implants (36)			
10 implants (1)			
Sections			
One (21/47) *			
Two (6/21)	0.47	(0.16–1.40)	0.179
Three (4/12)	0.68	(0.19–2.46)	0.560
Without Cantilevers	1.50	(0.51–4.39)	0.460
Maxilla (15/52) *			
Mandible (1/11)			
Retention	5.05	(1.88–13.59)	0.001
Cemented (7/34) *			
Maxilla (29)			
Mandible (5)			
Screwed (24/46)			
Maxilla (31)			
Mandible (15)			
Crown/Implant Ratio			
<1 (8/18) *			
Maxilla (17)			
Mandible (1)			
1 (18/49)	0.55	(0.16–1.64)	0.285
Maxilla (35)			
Mandible (14)			
> 1 (5/13))	0.68	(0.17–2.87)	0.606
Maxilla (8)			
Mandible (5)			
Opposing Dentition	0.82	(0.55–1.21)	0.32
Natural Teeth (30) *			
Acrylic/resin (2)			
Metal-Porcelain and Teeth (18)			
Metal-Porcelain (30)			

Total of mechanical-technical complications = 31. Univariate logistic regression analysis. Frequency in brackets (* = Reference category; OR = Odds ratio; ORCI = 95% Odds ratio Confidence Interval).

**Table 9:** Distribution of mechanical technical complications in the sample in relation to implant-based variables.

Implants (n = 558) (31/558)			
Variable	OR	(ORCI)	*p*
Site	1.04	(0.69–1.57)	0.828
Mandible (120) *			
Maxilla (438)			
Implant Area			
Incisal (150)			
Maxilla (119)			
Mandible (31)			
Canine (101)	0.99	(0.60–1.65)	0.986
Maxilla (77)			
Mandible (24)			
Premolar (167)	0.94	(0.60–1.46)	0.78
Maxilla (126)			
Mandible (41)			
Molar (140)	0.87	(0.55–1.39)	0.578
Maxilla (116)			
Mandible (24)			
Implant Diameter			
Narrow (< 4 mm) (5/82) *			
Standard (4–4.1 mm) (24/422)	0.98	(0.60–1.58)	0.937
Width (> 4.1 mm) (2/54)	0.44	(0.20–0.97)	0.043
Length	0.74	(0.39–1.40)	0.369
<10 mm (3/42) *			
> 10 mm (28/516)			
Connection	2.54	(1.06–6.10)	0.036
Internal (21/543) *			
External (10/15)			
Implant Brand	1.87	(1.27–2.75)	0.001
Straumann SLA Tissue Level (6/410) *			
Others (25/148)			
Astra Osseospeed (133)			
Biomet 3i Osseotite (15)			

Total of mechanical-technical complications = 31. Univariate logistic regression analysis. Frequency in brackets (* = Reference category; OR = Odds ratio; ORCI= 95% Odds ratio Confidence Interval).

**Table 10:** Life table on implant level.

Year Interval of Follow–Up	No. of Implants Follow–Up	No. of Failures	Survival Rate per Year Interval (%)	Cumulative Survival Rate (%)
0–1	558	0	100	100
1–2	558	1	99.8	99.8
2–3	557	0	100	99.8
3–4	557	0	100	99.8
4–5	557	0	100	99.8

**Table 11:** Life table on prostheses level.

Year Interval of Follow–Up	No. of Prosthesis Follow–Up	No. of Failures	Survival Rate per Year Interval (%)	Cumulative Survival Rate (%)
0–1	80	0	100	100
1–2	80	1	98.8	98.8
2–3	79	0	100	98.8
3–4	79	0	100	98.8
4–5	79	0	100	98.8

**Table 12:** Distribution of mechanical-technical complications.

Kind of Complication	Restoration Based	Patient Based	Screwed Prostheses N = 46	Cemented Prostheses N = 34	*p*
All Complications	31/80 (38.75%)	31/65 (47.69%)	26/46 (60.86%)	5/34 (14.71%)	0.001
Loss of the Access Plug	13/80 (16.25%)	13/65 (20.00%)	13/46 (34.78%)	0/34 0.00%	
Porcelain Fracture	10/80 (12.50%)	10/65 (15.38%)	7/46 (15.22%)	3/34 (8.82%)	0.396
Screw Loosening	4/80 (5.00%)	4/65 (6.15%)	3/46 (6.52%)	1/34 (2.94%)	0.467
Screw Fracture	1/80 (1.25%)	1/65 (1.54%)	1/46 (2.17%)	0/34 (0.00%)	
Implant Fracture	1/80 (1.25%)	1/65 (1.54%)	1/46 (2.17%)	0/34 (0.00%)	
Framework Fracture	1/80 (1.25%)	1/65 (1.54%)	1/46 (2.17%)	0/34 (0.00%)	
De-cementation	1/80 (1.25%)	1/65 (1.54%)	0/46 (0.00%)	1/34 (2.94%)	
